# Lymphoepithelioma-Like Carcinoma of the Lung Associated With Epstein-Barr Virus in a Caucasian Patient: A Case Report

**DOI:** 10.7759/cureus.30767

**Published:** 2022-10-27

**Authors:** Savvas Lampridis, Andreas Gkikas, Maria Chiara Pisciella, Giulia Salvatorelli, Davide Patrini

**Affiliations:** 1 Department of Thoracic Surgery, Guy’s and St Thomas’ NHS Foundation Trust, London, GBR; 2 Department of Thoracic Surgery, 424 General Military Hospital, Thessaloniki, GRC; 3 Department of General Surgery, The Hillingdon Hospitals NHS Foundation Trust, London, GBR; 4 Department of Thoracic Surgery, School of Medicine, University of L’Aquila, L’Aquila, ITA; 5 Department of Thoracic Surgery, University College London Hospitals NHS Foundation Trust, London, GBR

**Keywords:** lymphoepithelioma-like carcinoma, lung, epstein-barr virus, ebv, caucasian, case report

## Abstract

Primary pulmonary lymphoepithelioma-like carcinoma (LELC) is a rare tumour that is histologically identical to the lymphoepithelioma originally described in the nasopharynx. It mostly occurs in endemic regions in Asia, where it is associated with Epstein-Barr virus (EBV) infection. The incidence of primary pulmonary LELC is even lower in the Western population, in which an association with EBV is not typically observed. In this report, we present the case of a 78-year-old Caucasian woman who was diagnosed with EBV-positive pulmonary LELC following lingulectomy and histopathological examination of the tumour. Metastasis from the nasopharynx was excluded clinically and radiologically. Due to the absence of regional lymph node involvement, the patient did not receive adjuvant therapy. She remained free of disease at the one-year follow-up. Reporting cases of primary pulmonary LELC in non-Asian patients can help to elucidate the association with EBV infection and its potential implications for diagnosis, treatment, and follow-up.

## Introduction

Lymphoepithelioma-like carcinoma (LELC) of the lung is a rare tumour that is histologically identical to the lymphoepithelioma originally described in the nasopharynx. Primary pulmonary LELC has been mostly reported in Southeast Asia, where there is a strong association with Epstein-Barr virus (EBV) infection [1,2]. Its incidence is significantly lower in Western countries, making primary pulmonary LELC exceedingly rare in this population [2]. Furthermore, a relationship with EBV is not typically observed in non-Asian patients. Here, we present the case of an elderly Caucasian woman who was diagnosed with primary LELC of the lung associated with EBV.

## Case presentation

**Figure 1:**
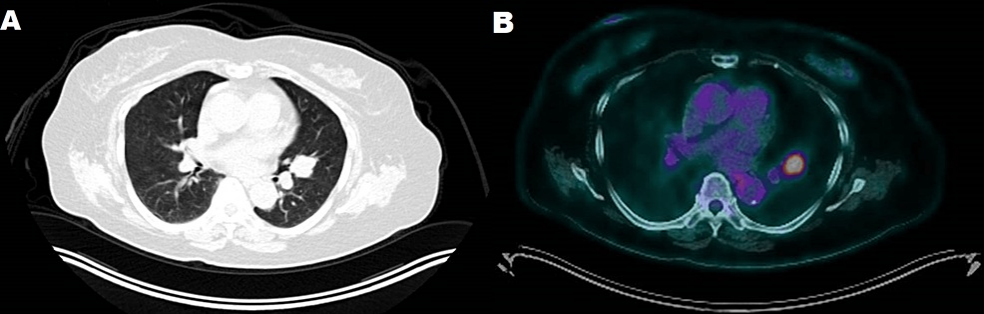
Radiological features of lymphoepithelioma-like carcinoma of the lung. (A) Computed tomography of the chest showing a solid, spiculated nodule in the lingula of the left lung. (B) Positron emission tomography with fluorodeoxyglucose integrated with computed tomography showing increased metabolic activity of the lung nodule.

A 78-year-old White female, born to British parents, presented with cough and dyspnoea on exertion, which had started three months prior and gradually deteriorated. Her medical history included obstructive sleep apnoea. She was a non-smoker, she had never visited Asia, and she had no relevant family history. Physical examination was unremarkable. Computed tomography (CT) of the chest demonstrated a solid, spiculated nodule in the lingula of the left lung measuring 25 mm in the greatest dimension (Figure 1A). Positron emission tomography with fluorodeoxyglucose integrated with CT revealed increased metabolic activity of the nodule (maximum standard unit value of 8.6) and the absence of mediastinal lymphadenopathy or distant metastasis (Figure 1B). The patient underwent video-assisted thoracoscopic lingulectomy with systematic lymph node dissection from stations 5, 6, 7, 10L, 11L, and 12L. She was discharged three days later after an uncomplicated postoperative course.

**Figure 2:**
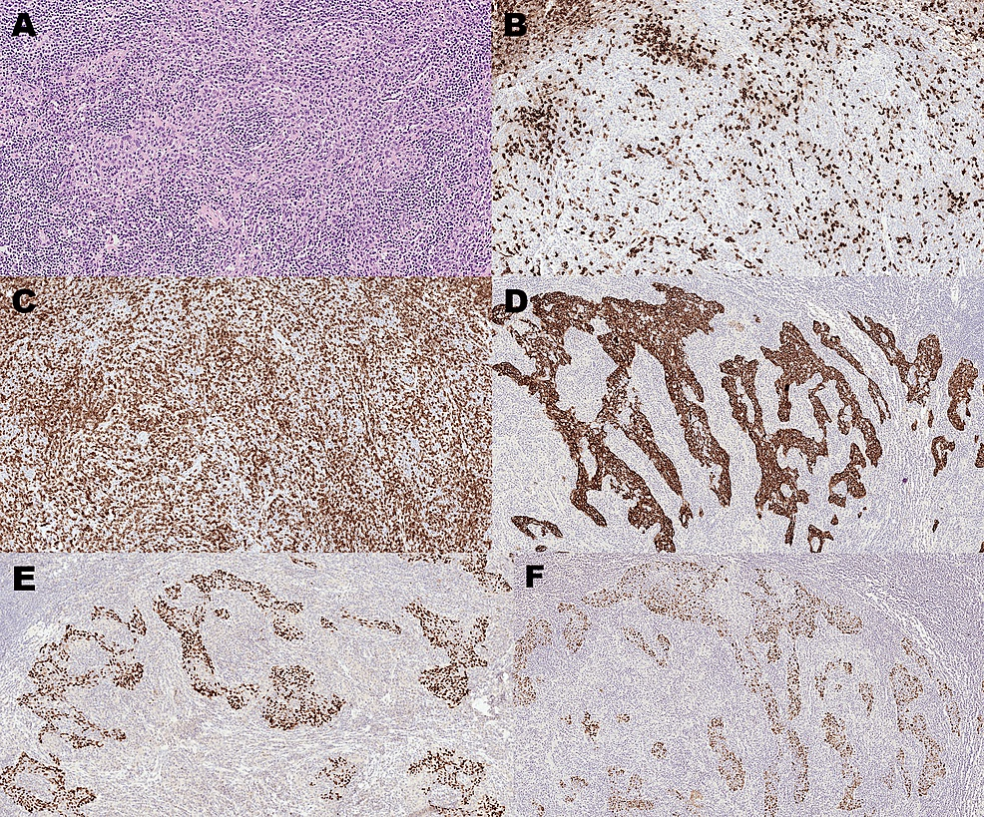
Histological characteristics of lymphoepithelioma-like carcinoma of the lung. (A) Haematoxylin and eosin stain shows pale epithelial islands admixed with numerous small lymphocytes (original magnification ×130). (B) CD20 immunohistochemical stain highlights scattered B-lymphocytes in the lymphoid component of the tumour (original magnification ×130). (C) CD3 immunohistochemical stain highlights abundant T-lymphocytes in the lymphoid component of the tumour (original magnification ×130). (D) MNF116 immunohistochemical stain highlights the epithelial component of the tumour (original magnification ×80). (E) The epithelial component of the tumour shows positive staining for p63 (original magnification ×80). (F) In situ hybridisation for Epstein-Barr virus-encoded small RNA shows a high proportion of positive epithelial cells (original magnification ×80).

Gross pathological examination of the lung specimen revealed a firm, white nodule measuring 25 × 20 × 15 mm. Microscopically, the tumour was relatively well circumscribed and permeated by a dense lymphoid infiltrate composed of a mixture of CD20-positive B-cells and CD3-positive T-cells (Figures 2A-2C). Within the mass, there were variably discrete islands of epithelial cells demonstrating mild-to-moderate cytologic atypia. The epithelial cells showed positive immunohistochemical staining for CKAE1/AE3, CK5, CAM5.2, MNF116, and p63 (Figures 2D, 2E). In situ hybridisation revealed the presence of EBV-encoded small RNA (EBER) in the nuclei of numerous neoplastic cells (Figure 2F). The morphological and immunophenotypical features of the tumour were consistent with pulmonary LELC. Because the pleura was free of tumour and the dissected lymph nodes showed no evidence of metastasis, the disease was staged as pT1cN0M0 (stage IA3), according to the eighth edition of the TNM classification for lung cancer. Nevertheless, we recognise that this classification may not apply to rare lung tumours, such as LELC.

Nasopharyngeal cancer was excluded by flexible nasopharyngoscopy performed by an otorhinolaryngologist, as well as CT of the head and neck. Because the tumour was in an early stage and completely resected, the patient did not receive further anticancer therapy. She has been followed up for one year with clinical history, physical examination, and CT of the chest. At her last follow-up, she had no complaints, and there was no evidence of disease recurrence.

## Discussion

In 1921, Schmicke as well as Regaud and Reverchon described an undifferentiated carcinoma of the nasopharynx with an intense lymphocytic component. This neoplasm has come to be known as lymphoepithelioma. LELC can originate from various anatomic sites, including the lung. Pulmonary LELC is a discrete histologic subtype of lung neuroendocrine tumours listed under the category of “other and unclassified carcinomas” in the 2015 classification of lung tumours by the World Health Organization. Since its first description by Bégin et al. [3] in 1987, approximately 300 cases have been reported in the English literature. The majority of these patients were Asians, mostly from Southern China, Taiwan, Hong Kong, and Japan. Only a small proportion of the reported cases have been described in non-Asian (Caucasian or Hispanic) patients (Table 1).

**Table 1:** Cases of primary pulmonary lymphoepithelioma-like carcinoma reported in non-Asian patients. ^a^ Detection of Epstein–Barr virus within tumour cells. ^b^ Stage is reported according to the eighth edition of the TNM classification for lung cancer, with the best possible approximation from the information provided. However, this classification may not apply to lymphoepithelioma-like carcinoma. CTx: chemotherapy; EBV: Epstein-Barr virus; F: female; ISH: in situ hybridization; LMP: latent membrane protein-1; M: male; NR: not reported; PCR: polymerase chain reaction; RTx: radiotherapy

Author	Year	Number of cases	Age (years)	Sex (number)	Smoking (number)	EBV detection^a^ (method)	Stage^b^ (number)	Treatment (number)
Butler et al. [4]	1989	3	56–72	M (1), F (2)	Yes (2), No (1)	No (ISH)	IA (2), IB (1)	Surgery (3)
Miller et al. [5]	1991	1	65	F	Yes	No (ISH)	IIB	Surgery
Ferrara et al. [6]	1995	2	64, 78	M (1), F (1)	Yes (1), No (1)	No (ISH)	NR	Surgery (2)
Wöckel et al. [7]	1995	1	47	F	NR	No (ISH, PCR)	IIIA	Surgery
Frank et al. [8]	1997	1	67	M	No	No (LMP)	IIB	Surgery + CTx
Barroso et al. [9]	2000	1	25	M	Yes	No (ISH)	IIIB	CTx
Castro et al. [10]	2001	6	49–75	M (4), F (2)	Yes (6)	No (ISH)	IA (4), IIB (1), IIIA (1)	Surgery (5), Surgery + RTx (1)
Jha et al. [11]	2002	1	57	F	Yes	No (LMP)	IA	Surgery
Morbini et al. [12]	2003	1	25	M	No	Yes (ISH, LMP)	IIIC	CTx + RTx
Bildirici et al. [13]	2005	1	66	M	Yes	No (LMP)	NR	NR
Hoxworth et al. [14]	2006	1	69	M	Yes	NR	IA	Surgery + CTx + RTx
Yener et al. [15]	2012	1	62	M	Yes	No (PCR)	IA	Surgery
Aktas et al. [16]	2017	1	69	M	Yes	No (ISH)	IA	Surgery
Fırıncıoğluları et al. [17]	2020	1	51	M	NR	Yes (ISH)	IIB	Surgery + CTx
Present case	2021	1	78	F	No	Yes (ISH)	IA	Surgery

In Caucasian patients, an association between EBV infection and pulmonary LELC is not typically observed, considering that such a relationship must be confirmed by the detection of EBV within tumour cells [2]. The presence of EBV in neoplastic cells can be demonstrated by immunohistochemistry for EBV-associated proteins (EBV nuclear antigen-2 and latent membrane protein-1), polymerase chain reaction for EBV DNA, as well as in situ hybridisation for EBV DNA or RNA. The latter is the most reliable method for the detection of latent EBV (a condition in which the virus predominantly exists) in paraffin sections [18]. Therefore, in situ hybridisation for EBER has become the standard investigation to demonstrate the potential association of pulmonary LELC with EBV and distinguish it from infection of circulating lymphocytes carried to the tumour site [1,2]. In the present report, EBER was detected in a high proportion of tumour cells. Moreover, the patient was born to White parents and had never travelled outside Europe, thereby excluding the commonly observed ethnic and geographic factors that appear to link EBV infection with pulmonary LELC.

Aetiological association between EBV and pulmonary LELC has been suggested but remains unclear. The finding of a single episomal form of EBV in tumour tissues suggests that EBV infection precedes the clonal expansion of the neoplasm, further supporting the oncogenic role of EBV [19]. However, the absence of EBV from LELC developing in other organs (e.g., liver, tonsils, urinary bladder, vagina, cervix) and the absence of morphological differences between EBV-associated and non-associated LELC suggest that tumorigenesis does not depend on EBV infection in a direct and exclusive manner [10,12].

Regarding its genetic profile, pulmonary LELC harbours only a low frequency of typical driver gene alterations that are found in common histologic types of non-small-cell lung cancer, including epidermal growth factor receptor (EGFR) mutation, anaplastic lymphoma kinase (ALK) rearrangement, Kirsten rat sarcoma viral oncogene homolog (KRAS) mutation, ROS proto-oncogene 1 (ROS1) rearrangement, and BRAF mutation. Furthermore, pulmonary LELC exhibits a high prevalence of copy number variations. Notably, chromosome 11 copy number gains may contribute to the pathogenesis of EBV-associated pulmonary LELC. Finally, mutations in epigenetic regulators, microsatellite instability, and loss of heterozygosity represent molecular disorders that may play a considerable role in the process of carcinogenesis.

The diagnosis of pulmonary LELC can be challenging in non-endemic regions due to its rarity and the lack of specific clinical and radiological findings. Therefore, the diagnosis is established by pathological examination of tissue specimens. This commonly occurs after surgical resection of the tumour, as in the case presented here. Cytological analysis of fine‐needle aspirates and bronchial brushing smears have also been reported to contribute to the diagnosis [20]. However, the accuracy of such techniques may be limited by the relatively scanty material and sampling errors. The differential diagnosis includes lymphoma and metastatic undifferentiated nasopharyngeal carcinoma. The distinction between lymphoma and LELC can be ascertained with a simple panel of immunohistochemical stains using keratin and leukocyte common antigens. In our case, the immunohistochemical profile of the neoplastic cells showed strong positivity for cytokeratins, favouring a diagnosis of LELC. Conversely, metastatic undifferentiated nasopharyngeal carcinoma is histologically indistinguishable from primary pulmonary LELC. For this reason, endoscopic examination and/or radiological imaging of the nasopharynx are necessary to exclude metastasis.

There is no consensus regarding the optimal treatment of pulmonary LELC. In the absence of prospective studies, investigators usually follow guidelines applied for the treatment of non-small-cell lung cancer. Nevertheless, pulmonary LELC appears to have equal or significantly better five-year survival compared to corresponding stages of non-small-cell lung cancer. Most reported cases of pulmonary LELC present early, and thus are amenable to surgical resection. In this case study, no adjuvant therapy was administered due to the small size of the tumour and the absence of regional lymph node disease. Postoperative chemotherapy and/or radiotherapy, however, have been used for locally advanced disease. In the presence of distant metastases, combination chemotherapy used for nasopharyngeal carcinoma (i.e., 5-fluorouracil, leucovorin, and cisplatin) has been proven an effective therapeutic regimen [2].

## Conclusions

Primary pulmonary LELC is an exceedingly rare tumour in non-endemic regions. Therefore, pathologists and clinicians must be knowledgeable of this entity to avoid misdiagnosis, which can trigger a cascade of negative outcomes. More cases should be reported to inform potential association with EBV infection in Caucasian patients and formulate appropriate strategies for diagnosis, treatment, and follow-up in this population.
